# Resolution of Exercise-Induced Syncope After Stenting of the Azygos Vein in a Dog with Segmental Aplasia and Azygos Continuation of the Levopositioned Caudal Vena Cava

**DOI:** 10.3390/ani15050722

**Published:** 2025-03-03

**Authors:** Viktor Szatmári, Henk van den Broek, Abraham N. Calero Rodriguez

**Affiliations:** Department of Clinical Sciences, Faculty of Veterinary Medicine, Utrecht University, Yalelaan 108, 3584 CM Utrecht, The Netherlands

**Keywords:** aneurysm, angiography, collapse, congenital vascular anomaly, echocardiography, episodic weakness, exercise intolerance, fainting, Holter, neurocardiogenic syncope, rivaroxaban, shock, stent, ultrasonography, retrocaval ureter, vasodepressor, vasovagal syncope

## Abstract

A 1-year-old Maltese–Poodle mixed breed dog was referred to the cardiology service because of severe exercise intolerance and daily exercise-induced syncopal episodes. Physical examination revealed no abnormalities. Echocardiography showed intermittent underfilling of the heart. Abdominal ultrasound examination revealed a segmental aplasia of the caudal vena cava between the kidneys and the liver, and azygos continuation of the cava. The azygos vein dorsal to the right kidney showed a severe aneurysm with stasis of blood. Computed tomographic angiography showed that the right crus of the diaphragm was probably responsible for the intermittent compression of the dilated azygos vein, which was thought to have subsequently led to insufficient venous return to the heart. The underfilled ventricles could not produce sufficient cardiac output, which caused the assumed cerebral hypoperfusion due to presumed systemic arterial hypotension. Under general anesthesia a self-expanding nitinol stent was implanted into the azygos vein at the level of the diaphragm. All clinical signs resolved immediately after surgery. The dog remained free from clinical signs (6 months after surgery). This is the first report that describes the successful treatment of this congenital vascular anomaly. Ultrasonography of the caudal vena cava should be performed in dogs with unexplained syncope.

## Simple Summary

In the present report, we describe the case of a 1-year-old dog with a very rare congenital anomaly of the abdominal blood vessels. A portion of the greatest vein of the body, which is responsible for returning the blood from the hindlegs and the belly to the heart, was missing due to a congenital developmental defect. A connection with another vein helped with the venous return. However, each time when the dog became excited or went for a walk, the venous return to the heart was insufficient to such a degree that multiple fainting episodes occurred every single day for several months. Advanced diagnostic imaging revealed that a compression of the “helping” vein by the diaphragm was responsible for the clinical signs. Implantation of a stent with a minimal invasive catheter technique prevented the vein from compression and resulted in immediate resolution of all clinical signs. The dog is free from any clinical signs 6 months after the surgery without any medication. This is the first report that describes this procedure for the treatment of this rare condition.

## 1. Introduction

The caudal vena cava (CVC) is the largest vein of the body, and it is responsible for venous return of blood to the heart from the caudal part of the body [1,2,3,4,5,6]. The CVC is formed from the common iliac veins at the level of the aortic trifurcation, it runs on the right side of the aorta in the retroperitoneum, then after a ventral divergence, it crosses the liver and the diaphragm, subsequently it enters the thorax in the caudal mediastinum, and it finally terminates in the right atrium. Several congenital anatomical anomalies of the CVC have been described, one of which is its segmental aplasia between the kidneys and the liver [1,2,3,4,5,6]. In this case, an anastomosis with the azygos vein guarantees that the blood from the interrupted CVC can still return to the right atrium. The right azygos vein is normally a much thinner vessel than the CVC, and it runs adjacent to the aorta in the retroperitoneum, through the diaphragm into the thorax, to finally enter the cranial vena cava. Congenital anomalies of the CVC have been rarely reported in dogs, presumably because most of them are clinically silent [1,2,3,4,5,6]. However, there are reports where segmental aplasia of the CVC with azygos continuation led to clinical signs, such as episodic weakness, renal failure, and pulmonary thromboembolism [1,2,3,4,5,6,7,8,9,10]. Our case report describes a dog with severe exercise intolerance and daily syncopal episodes, where the clinical and diagnostic findings were similar to those of a published case report [7]. The novelty of our case report is that stenting of the compressed azygos vein at the location where it crosses the diaphragm resulted in an immediate resolution of all clinical signs.

## 2. Case Presentation

A 1-year-old spayed female Maltese–Poodle mixed breed (Maltipoo) dog weighing 6.5 kg was presented to the cardiology service of the authors’ institution because of extreme exercise intolerance and daily exercise-induced syncopal episodes for about 5 months. Prior to the first syncopal episode, it was able to walk a kilometer with ease, but after the first heat, it could not walk farther than 100 m. In addition, excitement and exercise resulted in collapsing episodes. General functioning of the dog was otherwise normal, and no other problems were noticed.

**Figure 1:**
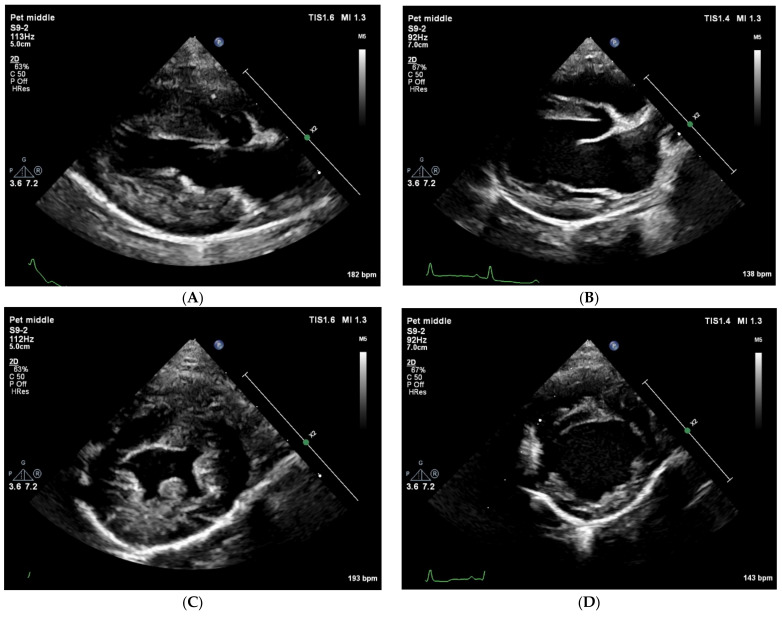
Transthoracic right parasternal two-dimensional grey-scale echocardiographic images at initial presentation (**A**,**C**) and 12 days later (**B**,**D**). All images show an end diastolic frame. Standard longitudinal four-chamber images (**A**,**B**) and standard short-axis images at the level of the papillary muscles (**C**,**D**) show at the initial presentation a severe underfilling of the left ventricle with severe concentric pseudohypertrophy, and normal wall- and lumen-dimensions 12 days later.

At presentation, the dog was bright, alert, and responsive with a body condition score of 4/9. The respiratory rate was 56 breaths/minute without effort. The femoral pulse quality was fair, and it was symmetrical with a rate of 144 beats/minute. Rectal temperature was 38.9 degrees Celsius. The buccal mucous membranes were pink, with a capillary refill time of 1 s. Cardiac auscultation revealed no murmur or arrhythmia. During a short walk within the clinic from the reception area to the cardiology room, the dog collapsed. During this episode, the femoral pulses could not be felt. Surface electrocardiogram was performed immediately, which showed sinus tachycardia with a rate of 200 beats/minute. Echocardiography showed a severe left ventricular underfilling with severe concentric (pseudo) hypertrophy (Figure 1), and a low peak aortic flow velocity measured from the subcostal view (Table 1). Blood tests were performed. Serum troponine-I concentration was within the reference range (0.01 ng/mL; reference interval [RI], <0.06 ng/mL) making a primary left ventricular hypertrophy unlikely. Basal cortisol concentration (323 nmol/L; RI, >55 nmol/L) was high enough to rule out hypocortisolism. The antigen test for *Angiostrongylus vasorum* infection was negative. N-terminal pro Brain Natriuretic Peptide (NT-proBNP) concentration was moderately elevated to 971 pmol/L. A 24 h Holter ECG was performed, which revealed sinus tachycardia during the two syncopal episodes that occurred during the registration. The heart rate during the registration varied between 32 and 265 beats/minute, and it was a sinus rhythm with occasional premature atrial complexes, maximal 3 beats/hour.

Twelve days after the initial visit a second consultation was planned. Blood pressure measurements on multiple extremities were performed to rule out aortic coarctation as a possible cause of the left ventricular concentric hypertrophy. Systolic blood pressure measured on the tail, a front leg, and a hind leg excluded aortic coarctation. Echocardiography was repeated and showed no abnormalities at all. Left ventricular internal and wall dimensions (Figure 1) as well as the peak aortic flow velocity were all within reference ranges (Table 1).

**Figure 2:**
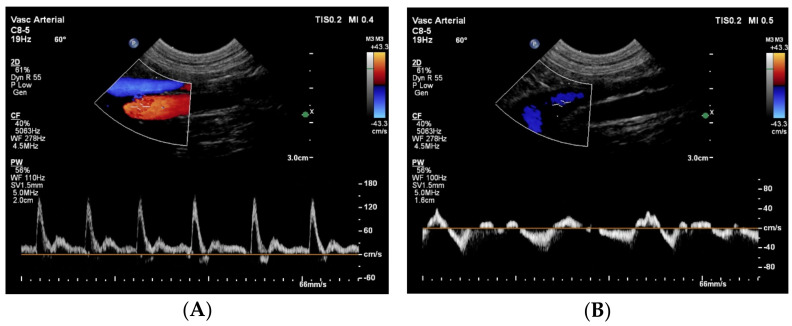
Duplex Doppler images of the abdominal aorta (**A**) and the caudal vena cava (**B**) caudal to the kidneys show normal spectral flow pattern in both vessels using pulsed-wave Doppler technique, but an abnormal anatomical localization, as the caudal vena cava (blue) is located to the left of the aorta (red). These images were obtained from the left lateral abdomen with the dog in right lateral recumbency. On both images left is cranial, right is caudal, top is left and bottom is right. With a normal aortic flow pattern, aortic coarctation was excluded.

Next, an abdominal ultrasound examination was performed to investigate the abdominal aorta and the CVC. The abdominal aorta showed normal pulsed-wave Doppler flow pattern, but unexpectedly, it was located to the right of the CVC (Figure 2).

**Figure 3:**
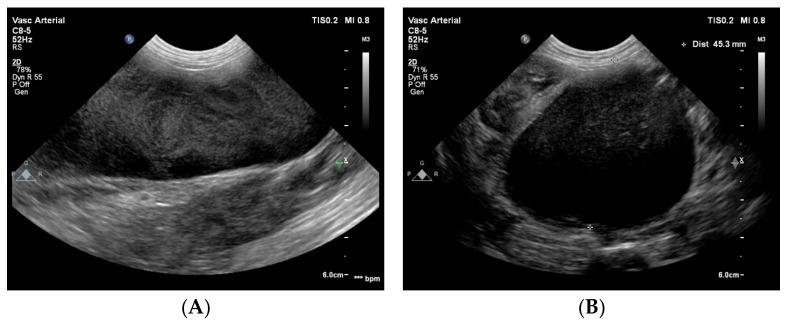
Two-dimensional grey-scale ultrasound images show an aneurysm of the azygos vein dorsal to the right kidney in longitudinal (**A**) and in cross section (**B**). In the vessel, spontaneous echo contrast is visible because of the blood stasis and sluggish flow. The images were obtained via the right lateral abdominal wall with the dog in left lateral recumbency. These findings suggest a severe venous obstruction downstream (i.e., cranial) to this location.

The CVC in the cranial abdomen showed an extreme aneurysm with a diameter up to 45 mm, with blood stasis to sluggish blood flow with spontaneous echo contrast in it (Figure 3). No thrombus was noted.

**Figure 4:**
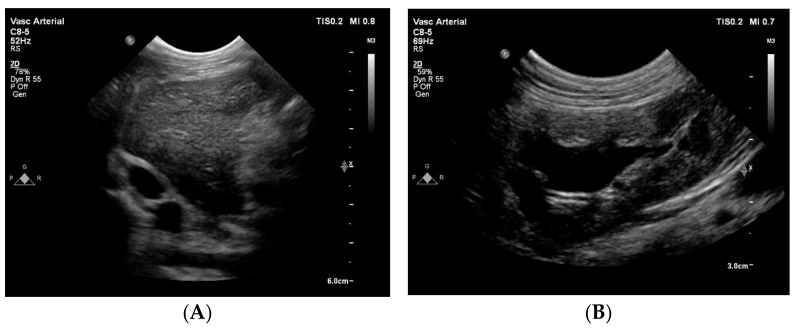
Two-dimensional grey-scale abdominal ultrasound images. (**A**) The great abdominal vessels in cross-section at the level of the liver obtained through an intercostal space from the right side with the dog in left lateral recumbency. To the left of the image is dorsal, to the right is ventral, to the top is right and to the bottom of the image is the left side of the dog. Dorsal and to the right of the aorta (round anechoic structure), an oval and equally large vascular structure is visible, compatible with an anatomical localization of a dilated (right) azygos vein. At the same time, no vessel can be seen at the location where the caudal vena cava is expected (ventral to the aorta, surrounded with liver tissue). (**B**) The left kidney displayed in a longitudinal image shows a moderately dilated renal pelvis (~1.7 cm). This finding is compatible with a unilateral ureteral compression or obstruction.

Cross-sectional ultrasound images through an intercostal space at the level of the liver showed no vascular structure at the normal anatomical localization of the CVC, but showed a vein adjacent to the aorta as an oval anechoic structure (Figure 4). The abdominal organs showed no abnormalities, except for a moderate dilation of the left renal pelvis (Figure 4). A computed tomography (CT) was recommended to better understand the anatomy of the congenital anomaly of the large abdominal veins. An obstruction of the venous return from the abdomen was suspected to be the cause of the syncopal episodes and the intermittent underfilling of the heart.

**Figure 5:**
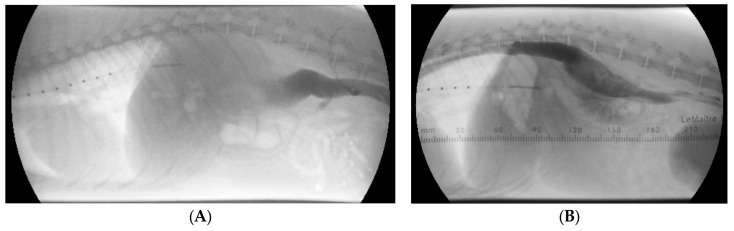
Non-selective venography before (**A**) and after (**B**) stenting of the azygos vein. With the dog in lateral recumbency, manual injection of iodinated contrast material was performed via the saphenous vein. A sizing catheter is located in the esophagus. (**A**) The caudal vena cava cannot be followed more cranial than the level of the first lumbar vertebra, and the dye does not reach the heart. (**B**) After stent implantation into the azygos vein at the level of the diaphragm, the dye reaches the heart. The contrast column flows through the aneurysmal dilatation of the azygos vein and reaches the intrathoracic part of the azygos vein. A stent guide is located under the dog.

Three weeks after the initial visit, a non-selective angiography and a CT scan were performed under general anesthesia. For premedication, butorphanol (0.6 mg/kg; Dolorex 10 mg/mL MSD Animal Health, Boxmeer, The Netherlands) was administered intravenously. For anesthetic induction and maintenance, propofol (PropoVet 10 mg/mL, Zoetis BV, Capelle aan den IJssel, the Netherlands) was administered intravenously as a variable rate infusion. For the non-selective venogram, an over-the-needle catheter (18 gauge, 1.3 × 45 mm, B-Braun, Melsungen, Germany) was placed into the right saphenous vein and 7 mL iodinated contrast material (1 mL/kg iobitridol 768 mg/mL, 350 mg iodin/mL, Xenetix 350, Guerbet, Villepinte, France) was injected manually. The contrast material in the CVC did not flow more cranial than the level of the first lumbar vertebra (Figure 5A).

A CT angiography of the abdomen and thorax was performed using a 64-slice sliding gantry CT scanner (Siemens Somatom Definition AS, Siemens Healthcare) 23 min after the non-selective angiography. The imaging protocol included precontrast and postcontrast acquisitions in the arterial, venous, and delayed phases. The patient was positioned in dorsal recumbency, and transverse abdominal images were obtained using the following parameters: 120 kVp, automatic tube current modulation (Care Dose) with a reference of 320 mAs, 0.5 s tube rotation time, 0.9 pitch, and a 512 × 512 matrix. Intravenous contrast administration was performed using a power injector, delivering iobitridol (Xenetix, Guerbet; 350 mg iodine/mL) at a dose of 2 mL/kg and rate of 2.0 mL/s with a maximum pressure of 203 PSI. Bolus tracking was applied using a region of interest placed in the abdominal aorta cranial to the celiac artery, with an attenuation threshold of 100 HU to trigger the arterial phase scan. The venous and delayed phase images were acquired 5 s and 60 s after the initiation of the arterial phase, respectively. Images were reconstructed using a soft tissue algorithm (B30f) with a 2 mm slice thickness. Multiplanar and three-dimensional (3D) volume-rendered reconstructions were generated and reviewed using a commercial Picture Archiving and Communication System (PACS; Agfa Healthcare Enterprise Imaging 8.2.2.050, Agfa N.V.).

**Figure 6:**
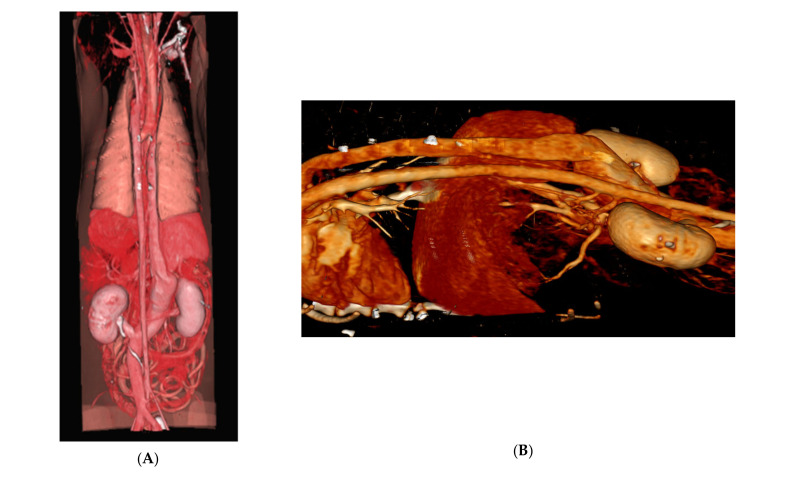
Volume-rendered three-dimensional projection of the computed tomographic angiography viewed from a dorsal (**A**) and left perspective (**B**). There is a large aneurysmal right medial cavo-right-azygos shunt. The left ureter courses dorsal to the caudal vena cava, consistent with a retrocaval ureter. The aorta has a normal appearance in its whole length (intra-abdominal and intrathoracic portions) with a uniform diameter throughout.

The CT scan showed that the entire abdominal portion of the CVC appeared dilated. Cranial to the kidneys the CVC was absent, and the renal and postrenal caval blood was directed through a large aneurysmal right medial cavo-right-azygos shunt with an isthmian connection to the right azygos vein. At the level of the aneurysm, the CVC was approximately 23 mm in diameter. Focal narrowing at the level of the right crus of the diaphragm (approximately 7 mm) and flattening of the azygos vein in between the right diaphragmatic crus and the thoracic wall at the level of 12th and 13th thoracic vertebrae were noted. The entire thoracic part of the right azygos vein appeared dilated (approximately 9 mm). Contrast uptake was homogeneous in the early and late venous phases, with no signs of intraluminal filling defects. In addition to the vascular abnormalities, a retrocaval course of the left ureter and a moderate dilation of the left renal pelvis were noticed. Three-dimensional rendering CT angiographic images are shown in Figure 6.

An intermittent compression of the dilated azygos vein by the right diaphragmatic crus was suspected to be the cause of the insufficient venous return during exercise and excitement. An intraluminal stent implantation was thought to resolve the clinical signs. Based on the dimeter of the CVC segment just caudal to the renal veins (12 mm), a bare metal self-expanding nitinol stent with a diameter of 14 mm and 50 mm length (Epic, REF H749 3905414507 0, Boston Scientific, Marlborough, MA, USA) was ordered for implantation.

For the minimal invasive catheter-based intervention, the dog was premedicated with a mixture of butorphanol 0.5 mg/kg, midazolam 0.4 mg/kg (Midazolam Eugia 5 mg/mL, Eugia Pharma, Valletta, Malta), ketamine 0.5 mg/kg (Narketan 100 mg/mL, Vetoquinol, Paris, France) and alfaxalone 1 mg/kg (Alfaxan 10 mg/mL, Jurox Limited, Dublin, Ireland), administered intramuscularly. General anesthesia was induced with propofol (intravenously) and maintained with propofol continuous rate infusion 0.25 mg/kg/min and remifentanil 0.15 mcg/kg/min (Remifentanil Mylan 2 mg, Mylan Pharmaceuticals Ltd., Dublin, Ireland). As antimicrobial prophylaxis, a single injection of cefazolin (22 mg/kg intravenously; Cefazolin Mylan, Eurofins Analytical Services Hungary Kft, Budapest, Hungary) was administered 20 min before incision.

**Figure 7:**
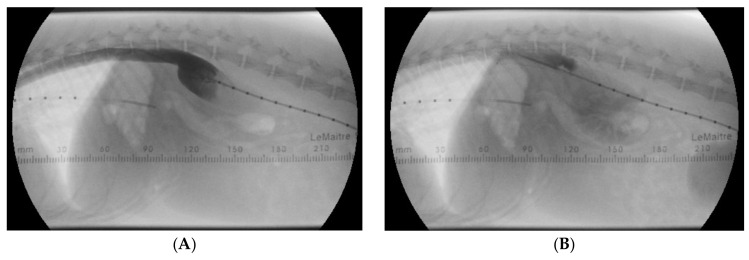
Angiographic images with the dog in right lateral recumbency before (**A**) and after (**B**) stent implantation. (**A**) Selective angiogram with the catheter tip in the aneurysmal dilated segment of the right azygos vein shows an abrupt reduction in the vessel’s diameter at the level of the diaphragm. (**B**) After the stent in place, and twenty seconds after performing a selective angiogram, contrast material is still present in the section of the azygos vein where the stent is located.

The dog was positioned in right lateral recumbency on the fluoroscopy table and a stent guide was placed underneath as well as a marker catheter in the esophagus. The skin over the left saphenous vein was surgically prepared. A skin incision over the left saphenous vein was made using a size 11 scalpel. With percutaneous puncture, a 7 French introducer (Braidin, 11 cm, APT medical, Shenzhen, China) was placed in the lumen of the left saphenous vein, and it was secured on the skin with a suture. Preoperative ultrasonography showed a vessel diameter of 2.6 mm at the puncture site, which accepted the introducer with an outer diameter of 3.00 mm (i.e., 9 French). After removal of the guidewire and the dilator, invasive pressure measurement via the side-port of the introducer was performed: a mean pressure of 6 mmHg was recorded. A straight vessel sizing catheter (5 French, 65 cm with 10 side holes, Infiniti Medical, Redwood City, CA, USA) was introduced into the CVC with a J-tip fixed core guidewire (InQwire Rosen, 0.035 inch, 260 cm, Merit Medical, South Jordan, UT, USA). After removal of the guidewire, an angiogram was performed with an injector (10 mL contrast material, 10 mL/sec with a pressure limit of 1000 PSI). The diameter of the intrathoracic azygos vein measured 9.3 mm (Figure 7). The chosen stent (14 mm in diameter) resulted in a 50% oversizing cranial to the obstruction. After removal of the angiographic catheter over the guidewire, the stent was introduced and released in a position so that it covered the stenotic region of the azygos vein.

**Figure 8:**
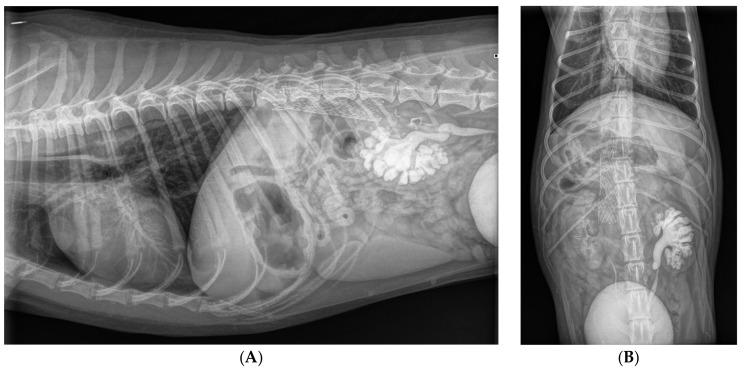
Radiographs in lateral (**A**) and in dorsoventral (**B**) projections made immediately after implantation of the stent in the right azygos vein. The stent is visible at the level of T12 to L1 vertebrae. As a result of renal excretion of the intravenously administered iodinated contrast material, the contrast is visible in the urinary bladder, both ureters and both kidneys. The left renal pelvis and the left ureter are moderately dilated as a result of an assumed compression of the retrocaval part of the left ureter.

After removal of the stent delivery system, the angiographic catheter was reintroduced over the guidewire. After removal of the guidewire, an angiogram was performed with machinal injection of 7 mL of iodinated contrast agent (10 mL/sec, 1000 PSI). The angiogram showed a good positioning of the stent, where approximately 1 cm of the stent was positioned in the aneurysm (Figure 5B and 7B). There was a fast clearance of contrast with a good cranially directed flow in the CVC and azygos vein (Figure 5B). However, some contrast remained in the segment of the azygos vein where the stent was positioned for at least 20 s (Figure 7B). Inspiration facilitated blood flow in the azygos vein into cranial direction towards the heart. Invasive pressure measurement via the side-port of the introducer showed a mean pressure of 8 mmHg. After removal of the catheter and introducer, the skin incision was closed with a purse string suture. An intravenous dose of heparin (100 IU/kg) was administered. Postoperative radiographs were made to document the position and shape of the stent (Figure 8).

The recovery from general anesthesia was uneventful and the dog was discharged on the same day with oral rivaroxaban (0.42 mg/kg q12h, Xarelto, Johnson & Johnson, New Brunswick, NJ, USA) prescribed for 6 months to reduce the chance of thrombus formation in the stent and the aneurysm of the right azygos vein. The skin suture was removed by the owner the next day.

Two days after the surgery, the owner reported via a telephone call complete resolution of the exercise intolerance and the syncopal episodes. A recheck physical and abdominal ultrasonographic examination 2 months after the surgery, showed no aneurysm of the azygos vein, but a persistent dilatation of the left renal pelvis. Six months after surgery the owner reported no clinical signs on the phone. Rivaroxaban administration was stopped 3 months postoperatively.

## 3. Discussion

The present case report describes the clinical and imaging findings of a dog with a congenital segmental aplasia and azygos continuation of the CVC. Exercise intolerance, episodic weakness, pre-syncopal and syncopal episodes arose from insufficient venous return to the heart caused by a compression of the azygos vein at the level where it crosses the diaphragm. Possibly shallow breathing, which accompanies exercise, does not create such a degree of negative intrathoracic pressure that could facilitate sufficient venous blood flow to the heart. In addition, the right crus of the diaphragm might reduce the diameter of the dilated azygos vein, with this further reducing its diameter. The reason why clinical signs appeared only at 8 months of age for the first time is hard to explain, because the venous anomaly had to be present from birth.

Despite of the fact that CT angiography failed to reveal a clear venous obstruction, the aneurysm of the azygos vein immediately caudal to the diaphragm suggests that there must have been a dynamic local obstruction to venous blood flow. Stenting of this region was thought to increase venous return to the heart. Following stent placement, clinical signs of exercise intolerance and syncopal episodes completely resolved, and the diameter of the abdominal CVC decreased, indicating a successful outcome and improved venous return. There is only one case report where a dog with the same venous anomaly was treated with an intraluminal stent, but the clinical signs in that case were attributable to renal failure [8]. In that case, renal failure resolved after stent implantation, and the dog remained in good clinical condition up to at least 3 years following stent placement.

Several routine diagnostic tests, such as echocardiography, Holter ECG, and blood tests failed to reveal the cause of syncope in the present dog. It is important to keep on looking for structural vascular anomalies in such cases before a diagnosis of vasovagal or vasodepressor syncope is presumed, which entities cannot be convincingly diagnosed in dogs. For this reason, we recommend performing a focused abdominal ultrasound examination specifically of the CVC in any dog where the cause of syncope has not been identified with physical examination, echocardiography, blood tests, and Holter ECG. Though the CVC is routinely imaged during echocardiographic examinations in dogs at the point where it crosses the diaphragm, in case of segmental aplasia of the CVC this part of the vessel as well as the hepatic veins look completely normal.

The increased plasma NT-proBNP concentration could be explained by physiologic left ventricular filling (i.e., stretch) after episodes of underfilling. The same etiology has been described for elevated NT-proBNP levels after pericardiocentesis in dogs with cardiac tamponade [12]. It is important to emphasize that a high NT-proBNP level does not mean that a heart disease is present.

Sinus tachycardia on the Holter ECG during the syncopal episodes confirmed that the episodic weakness was not caused by an arrhythmia. Sinus tachycardia is the result of a compensatory mechanism in case of systemic hypotension, and it is driven by the activation of the sympathetic nervous system [13].

Premature atrial complexes on the Holter ECG in the presented dog were interpreted as a clinically non-relevant coincidental findings. A possible cause for the premature atrial complexes can be atrial stretch due to the same etiology as it is described above for the elevated NT-proBNP level. However, atrial premature complexes have also been documented in Cavalier King Charles spaniels without any structural heart disease [14].

The levopositioned CVC is another congenital vascular anomaly, which is not related to the segmental aplasia of the CVC. However, it has been reported with retrocaval left ureter in dogs [15,16,17]. Levopositioned CVC was thought to be a clinically irrelevant coincidental finding in the present case.

In our case, compression of the left ureter was suspected to be the cause of a left-sided dilatation of the renal pelvis. This was a clinically silent, coincidental finding, and it was thought to be caused by the retrocaval ureter. The retrocaval ureter was thought to be another (third) unrelated congenital malformation in this dog, which has been described in a couple of other cases [16,17]. Dilation of the CVC might have contributed to the compression of the left ureter. Recheck ultrasonography 2 months after the stent implantation did not show resolution of the ureteral dilation, either because of ongoing compression, or because of the chronicity of the compression the dilation became irreversible.

For the current case, the decision was made to start anticoagulant prophylaxis treatment with rivaroxaban because of the stasis of blood that was apparent in the aneurysm of the azygos vein during the abdominal ultrasound examination and the diagnostic non-selective angiography before stent placement. Another reason for prophylactic anticoagulant treatment was the stasis of contrast within the newly implanted intravascular stent [18,19,20,21]. The reason for persistence of contrast in the stent, and it being only present there, is hard to clarify. A possible reason could be that the negative pressure during inspiration cannot increase the caliber of the stented segment of the azygos vein. The prophylaxis was prescribed for a duration of 6 months, because after this period the presumably completed neoendothelialization of the stent would prevent thrombus formation. The owner stopped rivaroxaban therapy after three months. Whether anticoagulant treatment was necessary for this dog at all is difficult to tell. In humans, stent implantation is not an indication for prophylactic anticoagulant or antiplatelet treatment. It is also hard to answer the question whether rivaroxaban is a better choice than clopidogrel to prevent thrombus formation in this situation. 

Limitation of the present case report is that six month follow-up might not be long enough to identify long-term complications of the stent implantation.

## 4. Conclusions

Stent implantation into the azygos vein resolved immediately the deteriorating clinical signs, which were thought to result from compression of the dilated azygos vein by the right diaphragmatic crus, in a dog with a segmental aplasia and azygos continuation of the CVC. Ultrasonographic examination of the abdominal CVC should be considered in every dog with unexplained episodic weakness and exercise-induced syncope before neurocardiogenic syncope is diagnosed.

## Figures and Tables

**Table 1:** Selected echocardiographic variables at the initial presentation and 12 days later without any intervention in between. At the first visit the left ventricular internal dimensions were markedly below the reference intervals, and the left ventricular wall thicknesses were above the reference intervals. These findings, together with the low peak aortic velocity, were suggestive for left ventricular underfilling. At the second visit all these variables were within the reference intervals.

	1st Visit	2nd Visit (12 Days Later)	Reference Interval
Left ventricular internal diameter in diastole	13.2 mm	25.8 mm	22.3–24.3 mm
Left ventricular internal diameter in systole	7.8 mm	15.9 mm	12.9–14.7 mm
Interventricular septum in diastole	10.1 mm	7.2 mm	6.5–8.0 mm
Left ventricular free wall in diastole	9.1 mm	6.3 mm	5.2–6.4 mm
Left ventricular internal diameter in diastole normalized for body weight	0.59	1.49	1.27–1.85 [11]
Left ventricular internal diameter in systole normalized for body weight	0.44	0.88	0.71–1.26 [11]
Peak aortic flow velocity	0.65 m/s	1.5 m/s	1.1–1.9 m/s

## Data Availability

Data are unavailable due to privacy restrictions.
